# Long-Term Abnormalities of Lipid Profile After a Single Episode of Sepsis

**DOI:** 10.3389/fcvm.2021.674248

**Published:** 2021-11-15

**Authors:** Nicholas Felici, Da Liu, Josh Maret, Mariana Restrepo, Yuliya Borovskiy, Jihane Hajj, Wesley Chung, Krzysztof Laudanski

**Affiliations:** ^1^Independence Blue Cross, Philadelphia, PA, United States; ^2^Department of Obstetrics and Gynecology, Shengjing Hospital of China Medical University, Shenyang, China; ^3^College Arts and Sciences, Drexel University, Philadelphia, PA, United States; ^4^College Arts and Science, University of Pennsylvania, Philadelphia, PA, United States; ^5^Corporate Informational Service, Penn Medicine, Philadelphia, PA, United States; ^6^Data Analytics Core, Penn Medicine, Philadelphia, PA, United States; ^7^Department of Nursing, Widener University, Chester, PA, United States; ^8^Society for HealthCare Innovation, San Francisco, CA, United States; ^9^Department of Anesthesiology and Critical Care, Hospital of the University of Pennsylvania, Philadelphia, PA, United States; ^10^Department of Neurology, Hospital of the University of Pennsylvania, Philadelphia, PA, United States; ^11^Leonard Davis Institute of Health Economics, Philadelphia, PA, United States

**Keywords:** sepsis, lipids, C-reactive protein, atherosclerosis, statin, gender, long-term, outcome

## Abstract

**Background:** Acute disturbances of the lipid profile are commonplace during acute sepsis episode. However, their long-term persistence has not to be investigated despite pivotal role of dyslipidemia in several comorbidities excessively noted in sepsis survivors (stroke, cardiomyopathy).

**Methods:** A total of 9,861 individuals hospitalized for a singular episode of sepsis between 2009 and 2019 were identified from electronic medical records. Lab measurements of total cholesterol (Tchol), high-density lipoprotein (HDL-c), low-density lipoprotein (LDL-c), very low-density lipoprotein (VLDL), triglycerides (TG), lipoprotein(a) [Lp (a)], apolipoprotein B (ApoB), and C-reactive protein (CRP). The data were examined as baseline values before sepsis, during hospitalization, and <3 months, 3–6 months, 6–12 months, 1–2 years, and more than 2 years from initial sepsis.

**Results:** Significant reductions in HDL-c (HDL_baseline_ = 44.06 vs. HDL_sepsis_ = 28.2; *U* = −37.79, *p* < 0.0001, Cohen's *d* = 0.22) and LDL-c serum levels were observed during and up to three months post sepsis, with females much less affected. In contrast, male subjects had derangement in HDL present for up to two years after a singular septic episode. Total cholesterol levels were slightly yet significantly elevated for up to two years after sepsis. TG were elevated up to one year [TG_baseline_ = 128.26 vs. TG_sepsis_ = 170.27, *t*(8255) = −21.33, *p* < 0.0001, Cohen's *d* = 0.49] and normalized. Lp(a) was elevated up to two years after initial episode [Lp(a)_baseline_ = 24.6 ± 16.06; Lp(a)_sepsis−2year_ = 8.25 ± 5.17; Lp(a)_morethan2years_ = 61.4 ± 40.1; ANOVA *F*_(2, 24)_ = 7.39; *p* = 0.0032]. Response to statin therapy was blunted in sepsis survivors for several years after sepsis resolution. Significant drop-out in prescription of statins and niacin after sepsis was observed. Serum high sensitivity C-reactive protein was elevated for up to five years after sepsis resolution (H [6;1685] = 502.2; *p* < 0.0001).

**Discussion:** Lipid abnormalities persisted long after the initial septic insult suggesting potential role in accelerating atherosclerosis and other abnormalities. In addition, sepsis seems to blunt statin effectiveness. Additionally, a significant and unexplained drop in statin use was seen in post-septic period.

**Conclusions:** Our study suggests that persistent derangements of lipid profile components for up to two years after sepsis may be associated with altered risk of atherosclerosis-related events among sepsis survivors.

## Background

Sepsis is a complex, heterogeneous disease state characterized by severe metabolic, neurological, immune, and hormonal disturbances (1–3). Long-term effects of sepsis are of mounting interest due to the increased survivorship and excessive long-term mortality (2, 4–6). Though etiology of excessive mortality among survivors is most likely complex and multifactorial, abnormal “wear and tear,” neurological abnormalities, metabolic reprogramming, or persistent immune dysregulation are potential causes (1–3, 7). The emergence of inflammatory or atypically activated monocytes (MO), inflammasome formation, endothelial activation, and cardiomyopathy are commonplace, during and, after acute sepsis (5, 8–12). Metabolic reprogramming during an acute episode of sepsis is often seen. Intuitively, these conditions may support the acceleration of atherosclerosis by induction of dyslipidemias. Lipid disturbances may lead to clinically relevant cardiovascular disease or susceptibility to subsequent infections (5, 13–16).

During the acute phase, abnormalities in serum high-density lipoprotein (HDL-c) and low-density lipoproteins (LDL-c), total cholesterol (Tchol), and triglycerides (TG) were reported (17–19). The time for return to optimal, or pre-insult, lipid profile in the aftermath of sepsis remains to be determined (20–24). Considering the pivotal role of lipid metabolism in homeostasis, their long-term abnormalities in sepsis survivors may explain excessive cardiac mortality and susceptibility to subsequent infections (2, 4, 13, 25, 26). Furthermore, abnormalities in the basic lipid profile composition may be compounded by sepsis-triggered lipoprotein modifications, serum level of lipoprotein a [Lp(a)] abnormalities, or apolipoprotein (Apo) B metabolism, contributing to atherosclerosis via different mechanisms than simple changes in composition of lipid profile components (16, 27–30). Furthermore, the persistence of inflammation measured as a serum C-reactive protein level may independently factor in atherosclerotic process (2, 20, 27, 31, 32).

Few studies have investigated duration and characteristics of long-term alterations in lipid profile after critical insult (33, 34). Changes in TG, HDL-c, LDL-c, ApoB, ApoAI, and ApoCIII levels were observed up to four months after initial infection was treated (34). Similar phenomena were described after cardiac surgery but the observation period was even shorter (35–37). In addition, murine animal data indicated progression of atherosclerosis in the aorta several months after resolution of the initial insult demonstrating acceleration of atherosclerosis in context of lipid profile abnormalities (38). However, establishing causation of atherosclerosis acceleration by singular septic episode in humans is challenging. Long-term studies of post-sepsis profiles are methodologically difficult as they must account for several confounders including smoking, diet, habits, physical activity, and others, while maintaining study subjects retention (3, 5, 11, 32, 39).

Here, we examined serum level lipid profile in patients surviving up to five years after singular episode of sepsis via electronic medical records (EMR). We focused on individuals where lipid profile components were measured prior to the sepsis, allowing for longitudinal analysis in a similar fashion to prior studies (34). CRP was assessed as a marker of inflammation and a independent risk factor for the emergence of cardiovascular disease (4, 13, 14, 31, 32, 40). We assessed the synthetic function of the liver, an essential organ in lipid metabolism. Finally, we accounted for gender, race, and intake of lipid lowering medications (31, 32).

## Methods

### Sample

The Institutional Review Board approved this study at the University of Pennsylvania (#832747).

**Figure 1:**
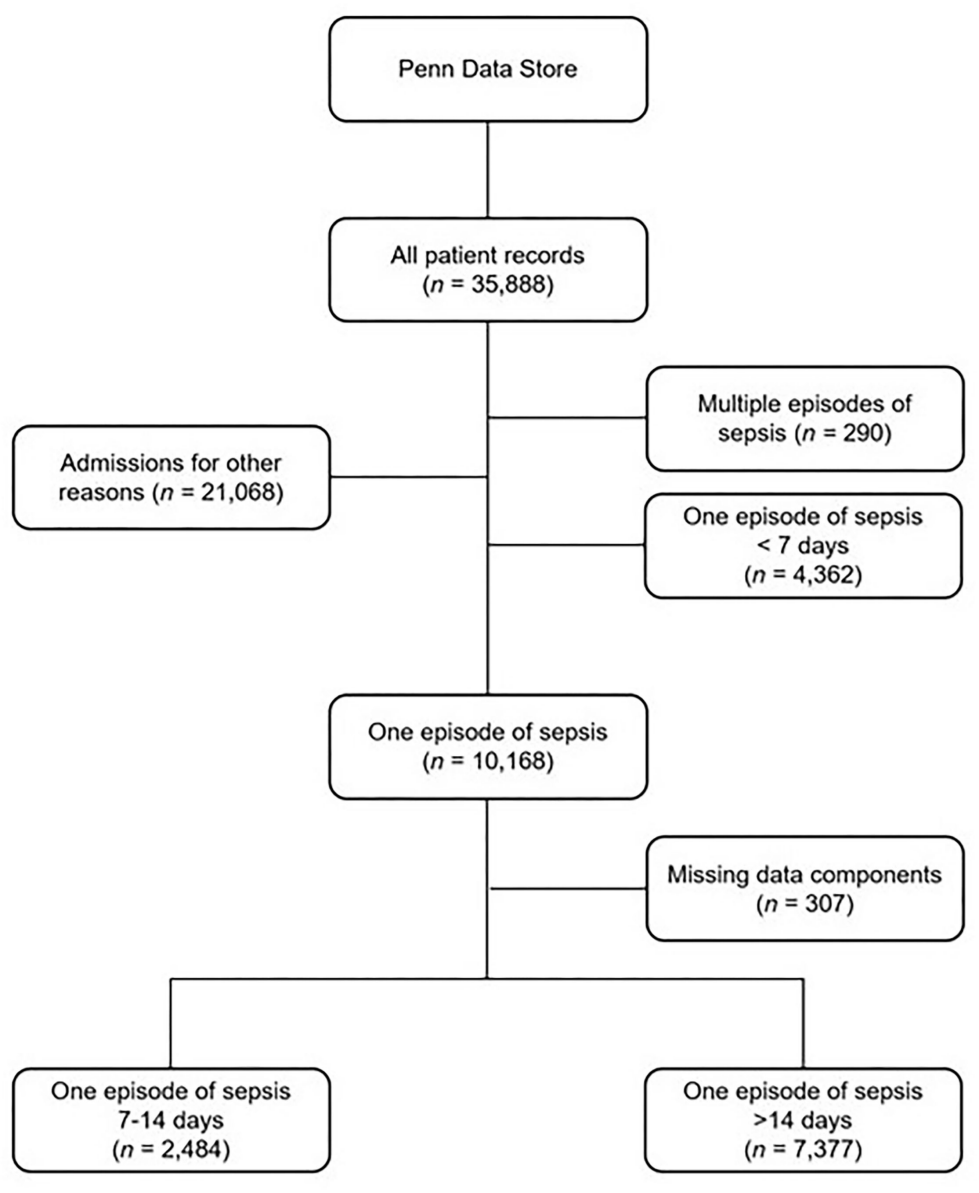
Processing of the electronic medical records (EMR) for the acquisition of the dataset.

Electronic medical records of individuals (*n* = 35,888) admitted to the intensive care units (ICU) at a large academic institution between January 2009 and January 2019 were screened using admission ICD-10 codes for sepsis (Appendix Table 1). These records were collected in three different hospitals across 10 different ICUs. Once patients with appropriate codes were identified, their medical records were examined one year before and up to five years afterwards (*n* = 14,818). Those who had two readmissions for sepsis were excluded from the analysis (*n* = 290). In addition, we excluded all hospitalizations with the length of stay shorter than seven days (*n* = 4,326) as most sepsis episodes last between 7 to 14 days (2, 3, 7). Final inclusion yielded a total of 9,861 individuals after eliminating records with missing data (*n* = 307) (Figure 1).

Lab values were obtained from the EMR. Lipid levels were measured in standardized and federally regulated labs. The patients' laboratory results were divided into the following categories: (1) baseline (up to one year before admission with sepsis), (2) during hospitalization, (3) < three months after discharge, (4) 3–6 months after discharge, (5) 6–12 months after discharge, (6) 1–2 years after discharge, and (7) more than one year after discharge (Appendix Table 2). The latter group was truncated at five years of observation period. Intervals were chosen to represent the acute pre-hospitalization baseline, acute disease onset, immediate recovery, rehabilitation, medium-length recovery, and long-term sequelae of sepsis (2, 3, 41, 42). Of note, average laboratory values were computed for those with multiple collections within the same period (Appendix Table 2). Finally, we eliminated outlier values, defined by three standard deviations above the mean, by removing them from the analysis (*n* = 32). This longitudinal design is modeled on a prior study (34).

### Definitions and Data Processing

Hospitalizations of sepsis episodes 7–14 days were considered a short sepsis episode. Hospitalizations longer than 14 days are classified as longer sepsis episodes (severe sepsis) (3, 7, 42). Sepsis episodes with a hospital stay below seven days were considered too short to represent a septic shock (2, 3, 42). All variables retrieved before admission for sepsis were considered baseline data. A random set of records of 50 individuals confirmed coding accuracy and primary diagnosis by manual chart review.

The definitions of cholesterol levels used for this study followed the guidelines established by the American College of Cardiology and the American Heart Association task force (20, 31, 32, 39, 43). The values that are considered abnormal this study are as follows: total cholesterol >200 mg/dL, HDL-c < 50 mg/dL (female) or < 40 mg/dL (male), LDL-c ≥ 100 mg/dL, VLDL-c > 30 mg/dL, triglycerides ≥ 175 mg/dL, Lp(a) ≥ 50 mg/dL, ApoB ≥ 130 mg/dL, and high sensitivity, or cardiac, CRP ≥ 2.0 mg/L (44).

Usage and dosage of medication was ascertained from EMR. Statin evaluated were as follows: Atorvastatin (Lipitor^®^), Fluvastatin (Lescol^®^), Lovastatin (Mevacor^®^, Altocor^®^), Pravastatin (Pravachol^®^), Pitavastatin (Livalo^®^), Simvastatin (Zocor^®^), Rosuvastatin (Crestor^®^). In addition, niacin and Niacin Extended Release were collected as well. Medications with frequency <0.2% at baseline were not included, considering the scarcity of the data, rendering statistical analysis unfeasible by way of being underpowered. Consequently, PCSK9 inhibitor, cholestyramine, ezetimibe (Zeta^®^), and fibrate were not included.

### Statistical Analysis

Shapiro-Wilk W-test and distribution plots were used to test the normality of distribution variables. Outlier values were defined as three standard deviations above the mean and were excluded from the analysis (*n* = 32). Parametric variables were expressed as mean ± SD and compared using Student's *t*-test. For non-parametric variables, median (M_e_) and interquartile ranges (IR) were computed with Mann-Whitney-U statistics, where the latter was employed to compare the study variables. Data groups were analyzed as independent groups, while longitudinal data were analyzed as paired, dependent samples. *d*-Cohen statistic was used to estimate the difference between means. A double-sided *p*-value of <0.05 was considered statistically significant for all tests. The Benjamin-Hochberg test was used to adjust *p*-values for multiple comparisons. *r*^2^-Pearson statistic was calculated to determine the correlation between the studied variables. Statistical analyses were performed using Statistica 11.0 (StatSoft Inc., Tulsa, OK). Graphs were generated using GraphPad Prism 8.4.2 (GraphPad Software Inc., San Diego, CA).

## Results

### Characteristics of the Sample

The characteristic of the studied population is presented in Table 1. Both genders were equally represented. Caucasian and Black races composed most of the population studied. Other races were not followed in the study considering overall low frequency in the dataset.

**Table 1:** Demographic characteristics of the studied sample.

**Characteristic**		**%**
age (mean ± SD)		62.5 ± 15.6
Gender	Male	58.4%
	Female	41.6%
Race	White	57.7%
	Black	29.5%
	Asian	2.3%
	Native American	0.05%
	Hawaii	0.08%
	Mixed	0.01%
	Other/ Unknown	9.91%
Sepsis Severity	Mild	32.9%
	Severe	67.1%

### Lipid Profile Trajectories Following a Single Sepsis Episode

#### High-Density Lipoprotein (HDL-c)

**Figure 2:**
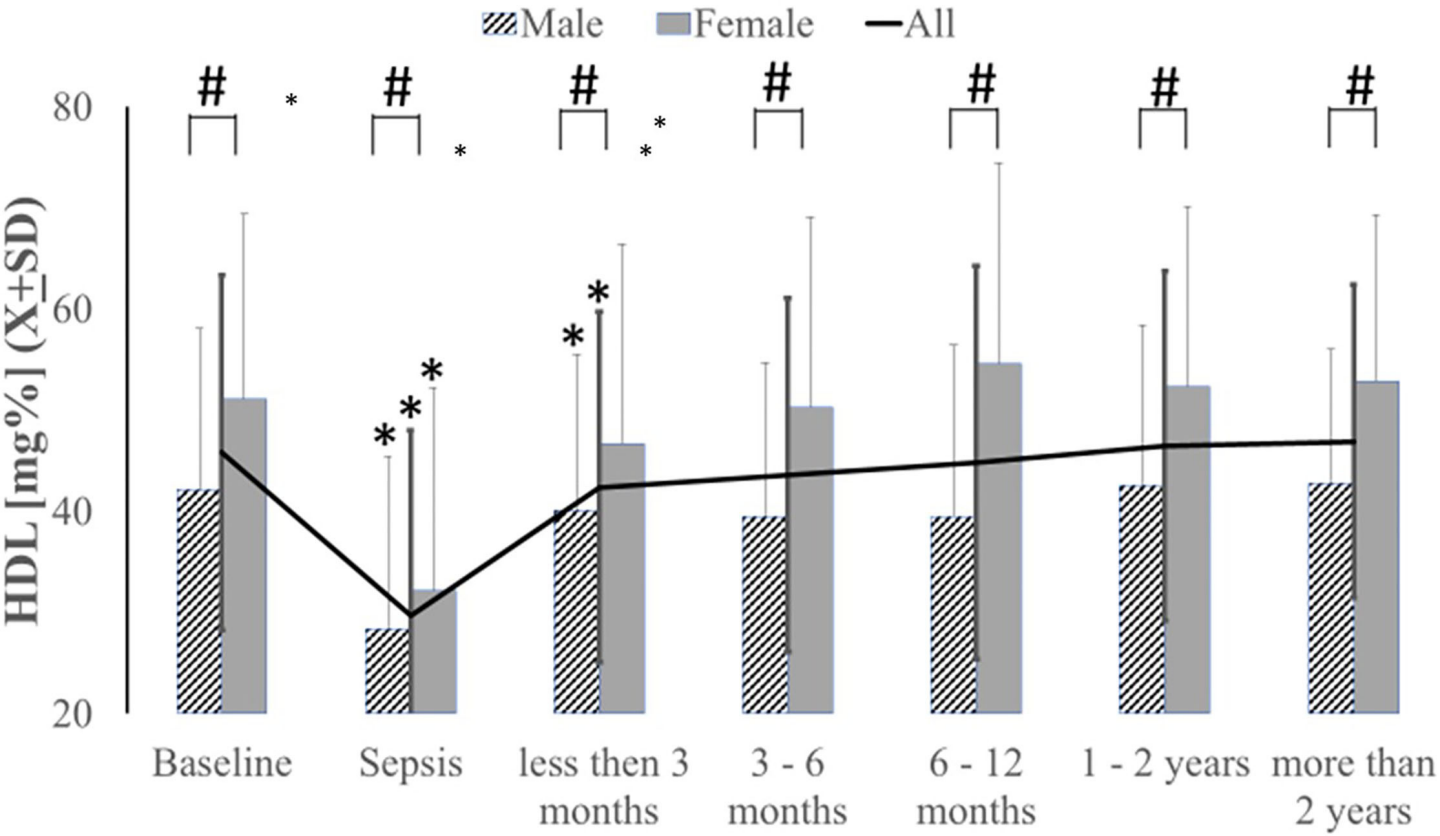
Serum HDL levels were decreased acutely during sepsis and slowly recovered within three months after sepsis with significantly less recovery female subjects. ^*^p < 0.05 as compared to baseline, # as compared between genders.

There was a significant reduction in the serum HDL-c level during sepsis compared to baseline (HDL_baseline_ = 44.06 *vs*. HDL_sepsis_ = 28.2; *U* = −37.79, *p* < 0.0001, Cohen's *d* = 0.22) followed by recovery after three months (Figure 2). Significant gender differences in serum HDL-c were present. The recovery of HDL-c in males was delayed up to two years, while females showed a tendency to have an increase in serum HDL-c level toward the end of the observation period (Figure 2). Patients with a severe episode of sepsis experienced a larger reduction of intra-sepsis HDL-c levels compared to patients with a nominal septic episode, albeit non-statistically significant [27.79 *vs*. 29.48, *t*(1495) = 1.91, *p* = 0.0561, Cohen's *d* = 0.12]. These differences did not persist after the episode of sepsis (data not shown). Black patients had significantly higher HDL-c levels at baseline and during the peri-sepsis period [30.85 *vs*. 27.19, *t*(1256) = 4.18, *p* < 0.0001, Cohen's *d* = 0.24] but these differences did not persist over time.

#### Low-Density Lipoprotein (LDL-c)

**Figure 3:**
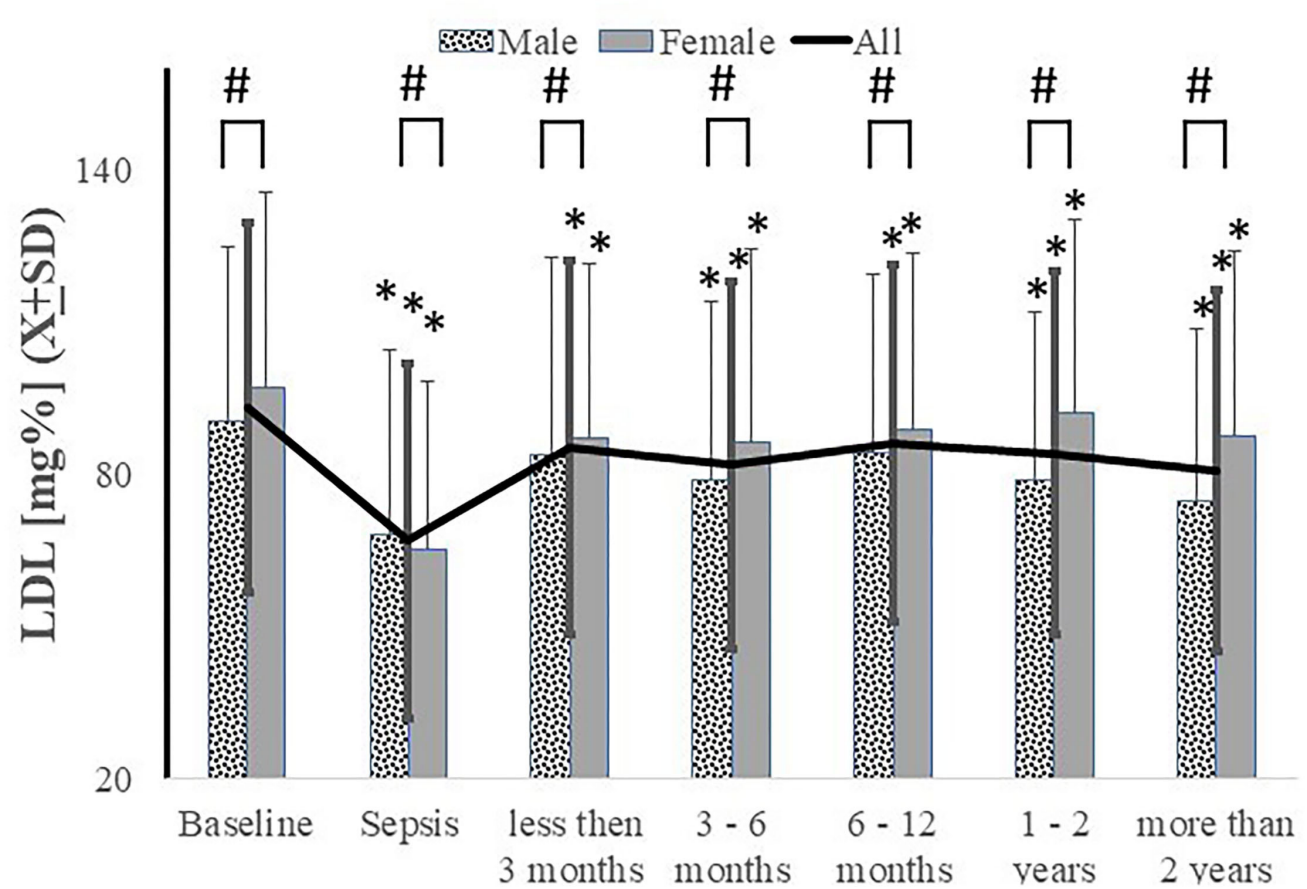
Serum LDL levels were decreased acutely during sepsis and never recovered to pre-sepsis level with significantly less recovery in male subjects. ^*^p < 0.05 as compared to baseline, # as compared between genders.

Decreased LDL-c level after an acute episode of sepsis persisted until the end of the study observation period (Figure 3). Females had significantly higher LDL-c levels than males at all times save during the sepsis episode (Figure 3). There were no significant differences in LDL-c levels based on sepsis severity (data not shown). Although black patients exhibited a higher serum LDL-c at baseline [92.99 *vs*. 88.34, *t*(2724) = 3.66, *p* = 0.0003, Cohen's *d* = 0.14], no significant differences were observed at >2 years post-sepsis [84.08 *vs*. 85.41, *t*(492) = −0.42, *p* = 0.6757, Cohen's *d* = 0.04].

#### Very Low-Density Lipoprotein (VLDL-c)

VLDL-c levels fluctuated slightly during the sepsis episode, but the changes were not statistically significant (H[422;6] = 6.97; *p* = 0.32]. Neither age (*r*^2^ = 0.067, *p* = 0.052) nor length of stay (*r*^2^ = 0.009, *p* = 0.423) correlated with post-sepsis VLDL-c levels. Sepsis severity [*F*_(1, 72)_ = 0.73, *p* = 0.40] and race [*F*_(3, 73)_ = 0.39, *p* = 0.76] did not correlated with post-septic changes in VLDL-c levels either.

### Total Cholesterol

**Figure 4:**
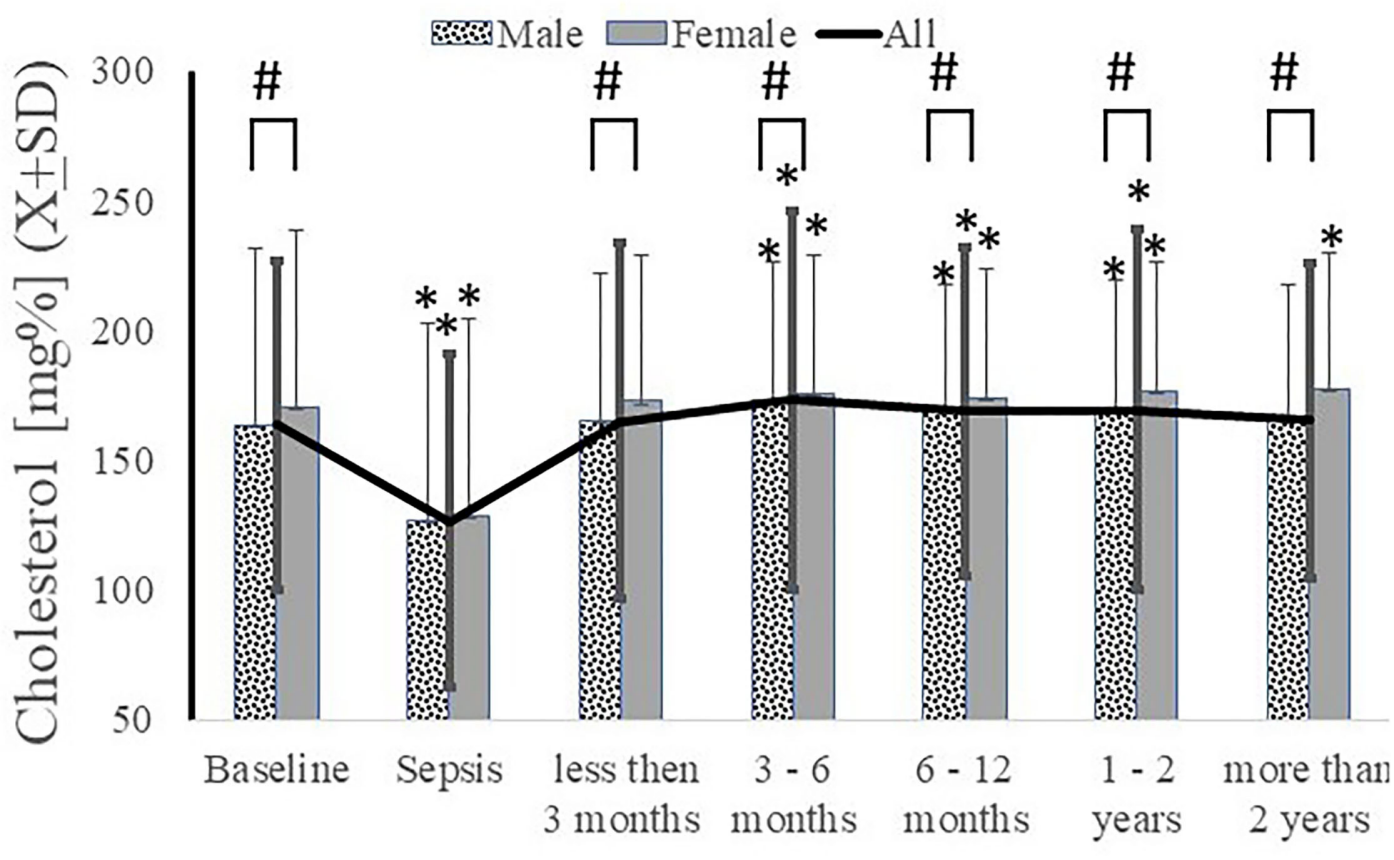
Serum cholesterol decreased acutely during sepsis and never recovered to pre-sepsis level with significantly less recovery in male subjects. ^*^p < 0.05 as compared to baseline, # as compared between genders.

There was a significant intra-sepsis reduction in serum total cholesterol levels. However, the levels quickly recovered and even increased beyond the pre-septic baseline (Figure 4). These differences were eliminated at >2 years following the episode when the data were analyzed as a group [157.64 *vs*. 160.25*, t*(2776) = −1.24, *p* = 0.2121, Cohen's *d* = 0.13] (Figure 4) or longitudinally [166.91 *vs*. 158.14, *t*(120) = 1.06, *p* = 0.2914]. Evaluation of serum total cholesterol levels revealed that females had slightly higher baseline total cholesterol levels at one-year post-sepsis (Figure 4). There were no significant differences in total cholesterol levels based on sepsis severity (data not shown). While there was no difference in baseline total cholesterol based on race, white patients had higher total cholesterol levels >2 years post-sepsis episode (data not shown).

### Triglycerides (TG)

**Figure 5:**
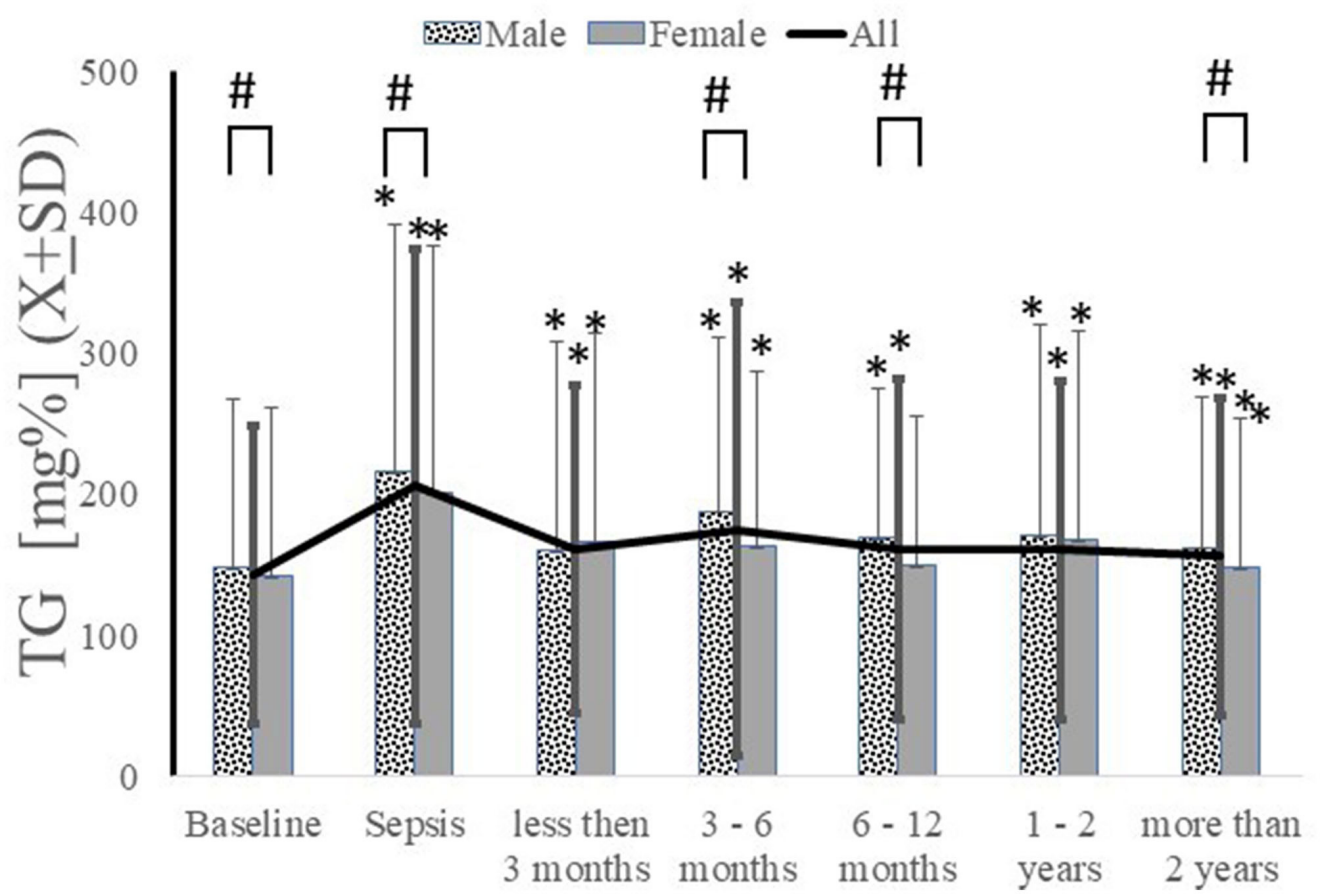
Serum triglycerides increased during sepsis and remained elevated in both genders or longitudinally. ^*^p < 0.05 as compared to baseline, # as compared between genders.

Intra-sepsis elevations in serum triglycerides were present [TG_baseline_ = 128.26 *vs*. TG_sepsis_ = 170.27, *t*(8255) = −21.33, *p* < 0.0001, Cohen's *d* = 0.49] and remained persisted at all post-sepsis time points when serial analysis was conducted (Figure 5). An increase in serum triglycerides was observed up to one-year post sepsis (Figure 5). The patient who had propofol administered during their sepsis episode exhibited a significantly higher level of triglycerides during the hospitalization (TG_onpropofol_ = 243.7 ± 220.27 *vs*. TG_offpropofol_ = 217.9 ± 196.41; *t*(8186) = 2.59; *p* = 0.0095). No differences in triglyceride levels were present at any time point after hospitalization concerning in-hospital propofol use [ANOVA *F*_(172300, 6)_ = 0.79; *p*=0.58]. While there were no significant differences in triglyceride levels during the peri-sepsis time points based on gender, males had significantly higher triglyceride levels beginning at three months post-sepsis (Figure 5). Patients with severe sepsis episodes also had significantly higher triglyceride levels than mild sepsis, but these differences disappeared beyond six months post-sepsis (data are not shown). There were significant differences in triglyceride levels based on race at all time points, with white patients having much higher serum levels (data not shown).

### Analysis of Lipid Profile

We dichotomized populations of patients based on their pre-insult profile as two groups: subjects with lipid profiles within recommended values and those with abnormal lipid profiles (13, 20, 31, 32, 39, 43). Those considered having recommended lipid profile presented total cholesterol levels < 200, HDL-c > 55, and LDL-c < 160 mg/dl (Pt_lipidfav_). These patients significantly decreased HDL-c levels while their serum cholesterol levels were elevated at six months post sepsis (Table 2) (Pt_lipid unfav_).

**Table 2:** Comparisons of long-term post-septic changes in lipid profile in patients with favorable and unfavorable lipid profile prior to a septic insult.

	**Patients with favorable baseline levels**				**Patients with unfavorable baseline levels**			
	***N***	**Baseline**	**After Sepsis**	***p***	***N***	**Baseline**	**After sepsis**	***p***
HDL	248	59.2 ± 13.0	56.7 ± 16.6	0.0099	319	33.9 ± 8.2	39.6 ± 11.9	<0.0001
LDL	334	72.6 ± 17.6	72.6 ± 26.9	0.9754	197	129.7 ± 32.1	105.8 ± 65.2	<0.0001
Total Cholesterol	212	127.7 ± 22.1	139.6 ± 35.7	<0.0001	218	201.6 ± 47.8	182.8 ± 71.7	<0.0001

*Favorable baseline levels were defined as HDL ≥ 45 mg/dL, LDL ≤ 100 mg/dL, and total cholesterol ≤ 160 mg/dL*.

Subjects with abnormal lipid profiles had the following lipid profile before sepsis total cholesterol levels > 240, HDL-c levels < 40, LDL-c levels > 160). In addition, patients with abnormal lipid profiles had decreases in serum levels of LDL-c, total cholesterol, and HDL-c at the long-term follow-up compared to those with recommended lipid profile numbers (Table 2).

### The Use and Effect of the Lipid-Lowering Agents in Sepsis Survivor

**Figure 6:**
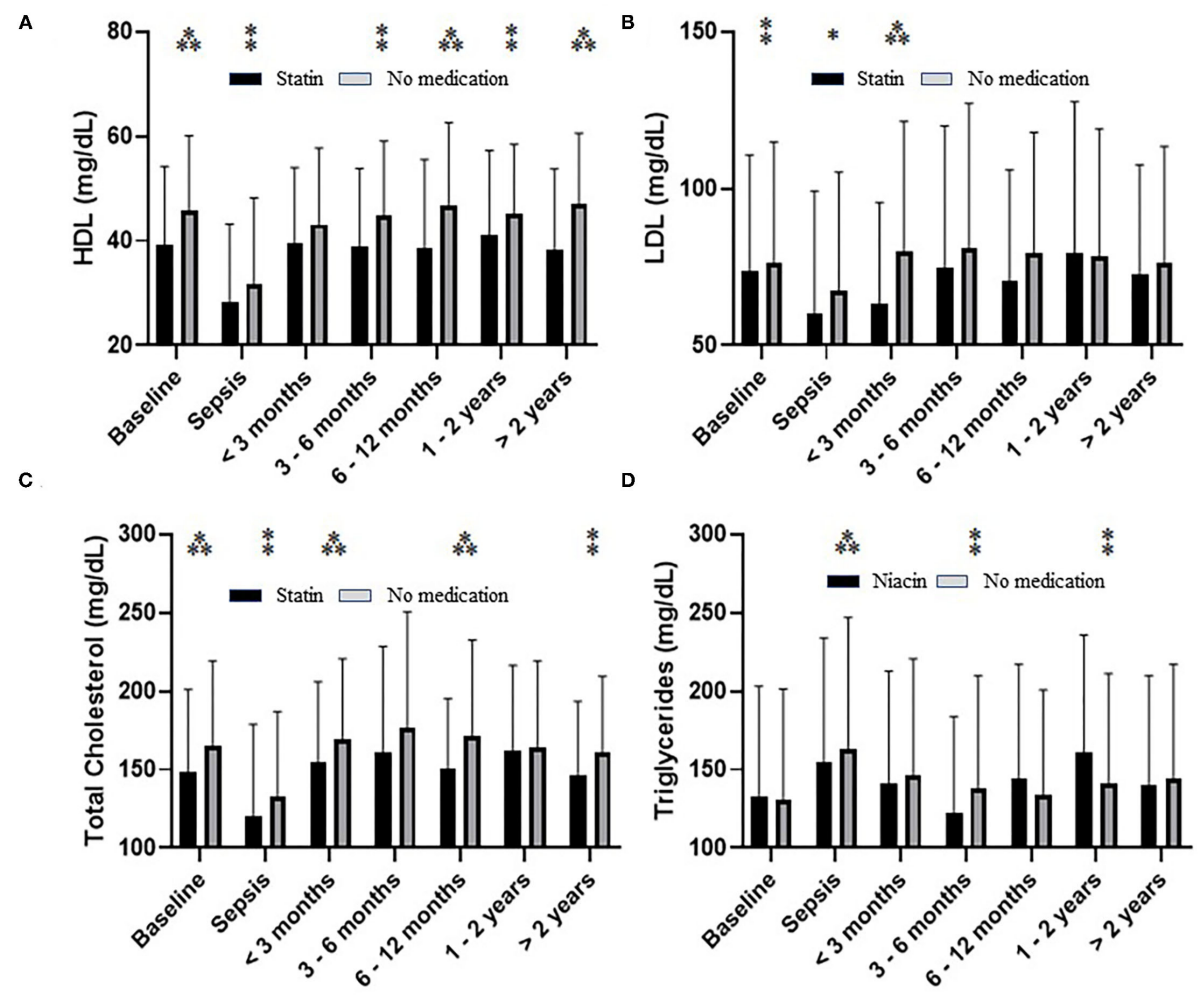
The effect of statin on serum level of HDL-c **(A)**, LDL-c **(B)**, total cholesterol **(C)**, as well as niacin on triglycerides **(D)** during and after sepsis. Significance based on pairwise comparison is denoted by ^*^(*p* < 0.05), ^**^(*p* < 0.01), or ^***^(*p* < 0.001) unless noted on figure.

Statin use was associated with significantly higher HDL-c serum levels than the control group (no statin use) (Figure 6A). In contrast, statin therapy's impact on LDL was abrogated at or after six months post sepsis (Figure 6B). The effect of statin therapy on total cholesterol was variable (Figure 6C). A similar pattern was seen regarding niacin's effect on triglyceride levels post-sepsis (Figure 6D). These data are based on a relatively small number of subjects due to a significant reduction in the number of individuals taking any of the lipid-lowering medications during recovery (Table 3). Interestingly, this reduction in frequency did not alter prescribing patterns as related to dosage (Appendix Figure 1).

**Table 3:** Frequency of statin and niacin use in each study interval.

	**Time points**						
	**Baseline**	**Sepsis**	**< 3 months**	**3–6 months**	**6–12 months**	**1–2 years**	**> 2 years**
Statin	37.5%	44.4%	12.8%	5.9%	6.0%	5.4%	4.5%
Niacin	2.0%	0.7%	0.1%	0.1%	0.1%	0.1%	0.1%

### Advanced Lipid Testing

Apolipoprotein B (Apo B) level carried grossly but insignificantly due to the small number of subjects [ANOVA *F*_(3, 36)_ = 0.84; *p* = 0.499]. Lp(a) showed significant variation with an initial decrease followed by a statistically significant increase at the 2 year mark [Lp(a)_baseline_ =24.6 ± 16.06; Lp(a)_sepsis−2year_ = 8.25 ± 5.17; Lp(a)_morethan2years_ = 61.4 ± 40.1; ANOVA *F*_(2, 24)_ = 7.39; *p* = 0.0032].

### Markers of Inflammation

**Figure 7:**
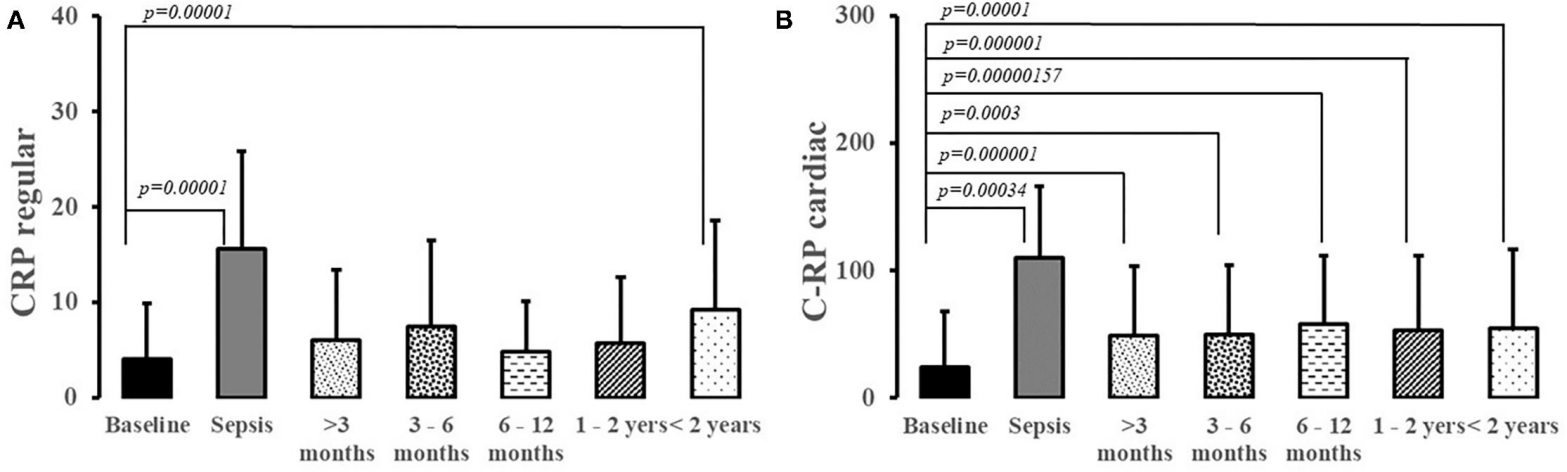
Regular **(A)** and cardiac C-Reactive Protein **(B)** levels in patients suffering from sepsis.

CRP level varied significantly during the observation period (H [663;6] = 178.8; *p* = 0.0001), but the elevation was significant only during sepsis and at two years (Figure 7A). However, an elevated CRP was observed for more than two years post-sepsis (H [1685;6)] = 502.2; *p* < 0.0001; Figure 7B). Sex and disease severity were not significant covariates in longitudinal changes of CRP post-sepsis (data not shown).

### Synthetic Function of the Liver

Serum protein and albumin levels exhibited depressed level during acute episode of sepsis but recovered at three months (Table 4). No differences in sex, race, and sepsis severity persisted long into the recovery of sepsis with respect to albumin and protein levels.

**Table 4:** Measurement of the synthetic function of the liver in studied time intervals.

	**Time points**						
	**Baseline**	**Sepsis**	**< 3 months**	**3–6 months**	**6–12 months**	**1–2 years**	**> 2 years**
Albumin	3.6 ± 0.8(3994)	2.7 ± 0.8(2612)	3.3 ± 0.6(1325)	3.6 ± 0.6(865)	3.6 ± 0.6(856)	3.8 ± 1.4(765)	3.8 ± 1.6(651)
Protein	6.5 ± 0.9(5356)	5.3 ± 1.0(8072)	6.3 ± 0.9(2629)	6.5 ± 0.9(1633)	6.6 ± 0.9(1629)	6.7 ± 0.8(1446)	6.7 ± 0.8(1080)

## Discussion

This is the first observational study exploring the long-term effects of a single episode of sepsis on the lipid profile components and CRP as markers of risk factors for acceleration of atherosclerotic disease (14, 31, 32). We analyzed close to 10,000 patients' EMR from the representative patient population treated in large academic healthcare system. Our analysis demonstrated an intriguing picture of persisting and significant derangements of various lipid profile components for up to two years after a single episode of sepsis. There was a significant decrease in serum HDL-c post sepsis level with rapid recovery in females but delayed in males. Lp(a) level was elevated up to two years after initial episode. High sensitivity CRP persisted through all observation periods. Triglyceride levels were increased initially after the episode of sepsis but normalized after one year. We did not quantify the clinical effect of these changes on the overall progression of atherosclerotic complications or subsequent risk of infection, but our study indicates a potential for clinical implications warranting further studies. Statistically, the lipid alterations seem to be moderate, as estimated by the *d*-Cohen statistics. However, the duration of these derangements, and their co-existence, was quite extensive. Taken at face value, similar changes are considered clinically significant (14, 21, 32, 45). It has been demonstrated that each 1 mg/dl increase in HDL-c level conferred a 2–4% decrease in cardiovascular risk per year (31, 32, 39). Interestingly, in large meta-analysis, the cumulative incidence of myocardial infarct, stroke, and heart failure in sepsis survivors was reported between 3 and 9% over a 5-year period (26).

A decrease in HDL-c level is linked to a profound increase in the incidence of atherosclerotic complications. Transient and persistent long-term decrease in HDL-c levels may increase cardiovascular risk, especially if it is coupled with an elevation in triglycerides but a decrease in LDL-c and cholesterol complicates the outcome of post-sepsis lipid profile (39, 43). The overall effect of these changes is difficult to estimate. Our study suggests that the changes are present and complex, but the lipid profile's overall effect warrants further prospective studies. These follow-up studies should include several other factors affecting the observed lipid profile abnormalities on the progression of atherosclerosis and susceptibility to infection (5, 33). The increased presence of inflammation, free radical species, lipid modification, and activation of atypical monocytes compounds the effects of dyslipidemia and may render the sepsis survivors particularly vulnerable to abnormalities in lipid profile post-sepsis (2, 9, 13, 14, 46–48). An animal study demonstrated that in ApE^−/−^ knockout animals, atherosclerotic plaques' burden was increased in long-term survivors suggesting that several concomitant derangements of the lipid profile are needed to trigger the progression of atherosclerosis (38). However, the authors did not study lipid profiles and attributed the changes to monocyte activation and metabolism alterations (9, 15, 34, 44, 48, 49).

The emergence of a persistent lipid profile is accompanied by aberrations in cardiac CRP or Lp(a), suggesting that changes in simple composition of lipid profile may not be the only factors accelerating atherosclerosis post-sepsis (44, 50). Lack of co-existing liver function abnormalities suggests reprogramming of both immune system and metabolism (1, 2, 12). Immune reprogramming is dependent more on the individual responsiveness, not the magnitude of the stimulus, but long-lasting and qualitative (15, 38, 40). Concomitantly, the severity of the disease measured by the length of stay was nearly not a significant factor in the duration or nature of the lipid changes. This is consistent with the notion of reprogramming, which is stimulus-independent once the septic insult reaches a threshold level (12, 13, 19, 25, 51). Metabolic reprogramming may also be responsible for the diminished effect of statin on lipid profile in sepsis survivors (6, 18, 19). Alternatively, diminished response to statins may be related to provider hesitancy secondary to the perceived risk of statin side effects (52). Our study suggests that the post-sepsis changes in lipid profile are present, but more controlled studies are needed. Such studies are particularly needed for individuals with nominal lipid profiles as they may be under-represented in our population. Also, such a population would be of particular interest to prevent atherosclerosis progression (32).

Interestingly, sepsis seems to affect females less than males (53). Concomitantly, lipid profiles in females recovered much faster than in their male counterparts. Gender's influence was dominant in the MONDO cohort, where long-term lipid abnormalities might have resulted in excessive mortality (45). In large cohort analysis, the risk of cardiovascular events was highest early after pneumonia, but excessive mortality secondary to cardiovascular events persisted for up to 10 years (33). Using propensity matching, an increased risk for myocardial infarction and ischemic stroke with a median observation duration of 6.7 years was seen (54). The excessive mortality was independent of sepsis severity, which is consistent with the idea of early reprogramming and threshold effect (1, 12, 55). Finally, bacteremia was not associated with a higher incidence of myocardial infarction, and stroke in a study limited to a one-year follow-up, suggesting that the initial insult must be severe enough to trigger a clinically relevant lipid profile (11).

One of the most puzzling observations was the dramatic drop in the use of lipid-lowering medication after an episode of sepsis. Study by Gupta et al. supports this observation as they observed a significant drop in prescribing statin secondary to provider's hesitancy (52). Perception of potential statin complications and patient frailty may inhibit the propensity of prescribers to engage in statin treatment even though in our study we did not see propensity to prescribe smaller doses of medications (32). This is consistent with the observation that providers have a skewed perception of risk and benefits (56). Alternatively, a lower incidence of statin prescription could be due to the lower LDL-c during recovery from sepsis, as patients would not fit the prescription criteria (32, 39). Still this should not be the reason for avoidance of statin, as they are recommended for secondary prevention after sepsis (2, 3, 7, 20, 31, 32, 42). In the studied healthcare system, clinicians are encouraged to follow the defined clinical pathways, but we cannot ascertain how much they follow system recommendations. One of the explanations is that patients are prescribed but not taking the medication themselves, but this should not affect provider prescription patterns (31, 32). Finally, patients may be filling the prescription with another provider, and we do not have access to that data. Finally, the influence of bias or error cannot be excluded.

The second puzzling finding is a somewhat blunted response to a statin. Statins were previously demonstrated to significantly lower cardiovascular event risk due to reduced LDL and total cholesterol as well as pleiotropic effect (32, 39). However, in our group, the patients with statin prescription had a significantly less pronounced effect on lipid profile despite being prescribed at similar dosages. The reason for diminished effectiveness is unclear, but metabolic reprogramming, mitochondrial dysfunction, or liver dysfunction may be the underlying cause (2, 11, 38, 56, 57). Lipid metabolism is mediated to a large degree by the liver which plays a critical role in the immunological response to sepsis and metabolic abnormalities (40). Specifically, liver fibrosis may emerge in sepsis survivors, and all lipid profile components may be severely affected while other function are preserved (57, 58). In our study, survivors recovered normal albumin and protein levels post sepsis. This suggests an altered metabolism of hepatocytes resulting in persistent abnormal lipid profile post-sepsis (1, 12, 51).

Our results aligned with prior findings and has several strengths. We demonstrated the lipid-lowering effect of statin on pre-septic patients' serum LDL in general (4, 14, 39). The decrease of LDL, cholesterol, and HDL during sepsis is frequently mentioned and correlate with disease severity (19, 59). Triglycerides were elevated in patients receiving propofol, as described before (60). Finally, we observed a significant effect of gender on lipid profile after time in individuals recovering from sepsis (31). In several cases, we were able to analyze the data longitudinally, reducing variability. Since this is the first observational study of its kind, there is a lack of comparative data except for three studies in different patient populations and a much shorter duration (35–37).

This is an observational, retrospective study of data obtained from medical records. EMR are prone to inclusion/exclusion bias, and missing records, measurement bias, and survivor bias, but these effects are difficult to judge and universal for these types of studies. There are inherent limitations to relying on ICD-10 data-based studies related to erroneous coding. We reviewed a small set of records to discover negligible coding problems, yet certain sepsis diagnoses were under-represented (23). We considered utilizing the sepsis laboratory parameters, but the lactic acid can be measured on different platforms resulting in a significant spread in the values. Utilizing the frequency of positive blood culture to diagnose sepsis is frequently linked to false-negative results or diagnosis of bacteremia, which is distinctive to sepsis (42). It is also unclear how the laboratory systems' evolution affects lipid profile measurements. There is some variability in the data secondary to introducing new diagnostic equipment, staff rotation, measurement drift. We decided against utilizing a control group since due to concerns regarding inherited bias and matching problems. Instead, wherever possible, we introduced longitudinal analysis with a pre-septic lipid profile as a control baseline.

Considering that patients with hypercholesterolemia would have blood drawn more frequently considering the AHA's recommendations, patients with nominal lipid profiles are likely to be under-represented in the study (32). There is also a bias including a subgroup of infectious diseases with particularly profound and distinctive effects on lipid profile, but the incidence of these illnesses was low in our data set (23, 30). Our analysis did not incorporate smoking, co-existence of elevated blood glucose levels, other co-morbidities affecting lipid profile, and compliance with medication regimens (3, 20, 25, 31, 32). These variables represent significant factors for the progression of cardiovascular disease and inflammation. Post-septic aberration in lipid profile may not be the only factor increasing cardiovascular risk or other morbidity factors. During recovery from sepsis, a significant percentage of patients developed abnormal or atypical monocyte traits (55, 61, 62). Some of these persistent post-sepsis MO characteristics involved activation of M-CSF and PU.1, factors playing an essential role in the emergence of foamy cells and persistent inflammation (8, 48, 49, 63). Furthermore, sepsis itself may cause myocardial injury, dilated cardiomyopathy, adrenergic overstimulation leading to unfavorable outcomes in the short- and long-term (4). Finally, analysis of lipoprotein needs to be done in the context of genetic makeup as the allele variability is a significant factor in determining atherosclerosis progression. Finally, our study was under-represented for Asian, and other races.

Despite its limitations, our exploratory study demonstrates a potential long-term effect of sepsis on the lipid profile and a diminished effect of statins on survivors. The results are intriguing enough to warrant further studies utilizing longitudinal observation and clinical endpoints of atherosclerosis (sudden death, myocardial infarction, cerebrovascular events).

## Conclusions

The novel finding of this study is the persistent derangements of LDL, total cholesterol, Lp(a), and high sensitivity (cardiac) CRP up to two years after sepsis. However, it may be associated with several abnormalities seen in sepsis survivors and may put patients at risk of developing an atherosclerotic condition. Future studies should address the long-term cardiovascular outcomes among sepsis survivors and re-address the study result with another database.

## Data Availability Statement

Data set can be released after IRB approval upon reasonable request. Requests to access these datasets should be directed to Krzysztof Laudanski, klaudanski@gmail.com.

## Ethics Statement

The studies involving human participants were reviewed and approved by the Institutional Review Board approved this study at the University of Pennsylvania (#832747). Written informed consent for participation was not required for this study in accordance with the national legislation and the institutional requirements.

## Author Contributions

NF: conceptualization, formal analysis, writing—original draft, and writing—review and editing. DL, JM, and JH: formal analysis and writing—original draft. YB: data curation. WC: formal analysis. KL: conceptualization, formal analysis, data curation, and writing—original draft. MR: visualization and manuscript review. All authors reviewed the final version of the manuscript and agreed to its publication.

## Funding

This work was supported by the NIH NIGMS Award 5K23GM120630-02 and the corresponding author's own funds.

## Conflict of Interest

The authors declare that the research was conducted in the absence of any commercial or financial relationships that could be construed as a potential conflict of interest.

## Publisher's Note

All claims expressed in this article are solely those of the authors and do not necessarily represent those of their affiliated organizations, or those of the publisher, the editors and the reviewers. Any product that may be evaluated in this article, or claim that may be made by its manufacturer, is not guaranteed or endorsed by the publisher.

## Abbreviations

MO: monocytes
statin: HMG-CoA reductase inhibitor
Me: median
IR: interquartile ranges
HDL: high-density lipoprotein
LDL: very low-density lipoprotein
Pt_lipidfav_: subjects with favorable lipid profiles
ApoB: apolipoprotein B
CRP: C-reactive protein
TG: triglycerides
GGTP: gamma glutamyl transferase
LPS: lipopolysaccharide
CVD: cardiovascular disease.
