# Supplementary material for: Long-Term Abnormalities of Lipid Profile After a Single Episode of Sepsis
Source: Front Cardiovasc Med. 2021 Nov 15;8:674248. doi: 10.3389/fcvm.2021.674248 (PMC8634493; doi:10.3389/fcvm.2021.674248)

Appendix

Appendix Table 1.

ICD-10 admission codes extracted as admission codes.

Code	Description
**A41.9**	Sepsis (generalized) (unspecified organism)
**R65.20**	Sepsis with organ dysfunction(acute) (multiple)
**R65.21**	Sepsis with septic shock
**R42.7**	Actinomycotic sepsis
**A39.1**	adrenal hemorrhage syndrome (meningococcal)
**A41.4**	Anaerobic sepsis
**A22.7**	Bacillus anthracis
**A23.9**	Brucella - see also Brucellosis
**B37.7**	Candida sepsis
**T85.79**	Sepsis due to device, implant or graft
**T82.7**	Sepsis due to arterial graft NEC
**T85.79**	Sepsis due to breast(implant)
**T85.79**	Sepsis due to catheter NEC
**T82.7**	Sepsis due to dialysis(renal)
**T85.71**	Intraperitoneal sepsis
**T82.7**	Sepsis due to infusion NEC
**T85.735**	Sepsis due to Spinal (cranial) (epidural) (intrathecal) (spinal) (subarachnoid) (subdural) catheter
**T83.511**	Sepsis due to urethral indwelling catheter
**T83.518**	Sepsis due to urinary tract infection
**O08.82**	Shock following ectopic or molar pregnancy
**T84.7**	Sepsis due to electronic(electrode) (pulse generator) (stimulator) bone device
**T84.69**	Sepsis due to internal fixation device of another site, initial encounter
**T85.79**	Gastrointestinal (bile duct) (esophagus)
**T85.732**	neurostimulator electrode (lead)
**T83.69**	Genital
**T82.7**	Heart NEC
**T82.6**	Valve (prosthesis)
**T82.7**	Graft
**T85.79**	Ocular (corneal graft) (orbital implant)
**T84.7**	Orthopedic NEC
**T85.79**	Specified NEC
**T82.7**	Vascular devices
**T85.730**	ventricular intracranial (communicating) shunt
**A41.81**	Enterococcus
**A26.7**	Erysipelothrix(rhusiopathiae) (erysipeloid)
**A41.5**	Escherichia coli (E. coli)
**A28.2**	Extraintestinal yersiniosis

**A41.9**	Gangrenous
**A54.86**	Gonococcal
**A41.5**	Gram-negative (organisms)
**A41.3**	H. influenzae
**B00.7**	Herpes viral
**K65.1**	Intra-abdominal
**A32.7**	Listeria monocytogenes
**T81.49**	Localized - code to specific localized infection in operation wound
**A24.0**	Pseudomonas mallei
**A24.1**	Melioidosis
**A39.2**	Acute meningococcemia
**A39.3**	Chronic meningococcemia
**A41.01**	Methicillin-Sensitive Staphylococcus Aureus (MSSA)
**P36.5**	Anaerobes NEC
**P36.4**	Escherichia coli (E. coli)
**P36.30**	Staphylococcus
**O85**	puerperal, postpartum, childbirth(pelvic)
**A02.1**	Salmonella(arizonae) (cholerae-suis) (enteritidis) (typhimurium)
**R65.20**	Severe sepsis without septic shock
**A03.9**	Shigellosis, unspecified
**A41.89**	Other specified sepsis
**A41.2**	Staphylococcus, staphylococcal
**A41.01**	Aureus (methicillin susceptible) (MSSA
**A41.02**	Aureus (methicillin resistant) (MRSA)
**A41.1**	Coagulase-negative Staphylococcus
**A40.9**	Streptococcus, streptococcal
**A40.1**	Streptococcus agalactiae
**A40.0**	Group A Streptococcus
**A40.1**	Group B Streptococcus
**A41.81**	Group D Streptococcus
**O86.04**	obstetrical procedure
**A40.3**	Streptococcus pneumoniae
**A40.0**	Streptococcus pyogenes
**J95.02**	Tracheostomy, stoma
**A21.7**	Tularemia
**A20**	Yersinia pestis, unspecified

Appendix Table 2.

Number of repetitive values for each measurement in predetermined time intervals

		Time Points					
	**Repetitive Values**	**Baseline**	**Sepsis**	**< 3 months**	**3 - 6 months**	**6 - 12 months**	**1 - 2 ye**
**HDL**	1-5	2386	1520	419	347	457	557
	6-10	390	2	2	2	3	4
	11-20	180	1	1	1	1	1
	> 20	40	0	0	1	2	0
**LDL**	1-5	2315	1469	402	337	428	530
	6-10	392	2	2	2	3	6
	11-20	174	1	1	1	1	1
	> 20	48	0	0	1	2	0
**Total Cholesterol**	1-5	2173	1867	458	383	456	544
	6-10	182	3	29	5	23	20
	11-20	54	1	3	1	2	5
	> 20	25	0	0	1	2	0
**Triglycerides**	1-5	2673	4277	756	470	535	611
	6-10	454	474	54	23	43	34
	11-20	235	151	12	7	13	16
	> 20	103	26	5	3	2	2

Appendix Figure 1.

Frequency of different dosin og atorvastatin before and after sepsis did not change significantly.

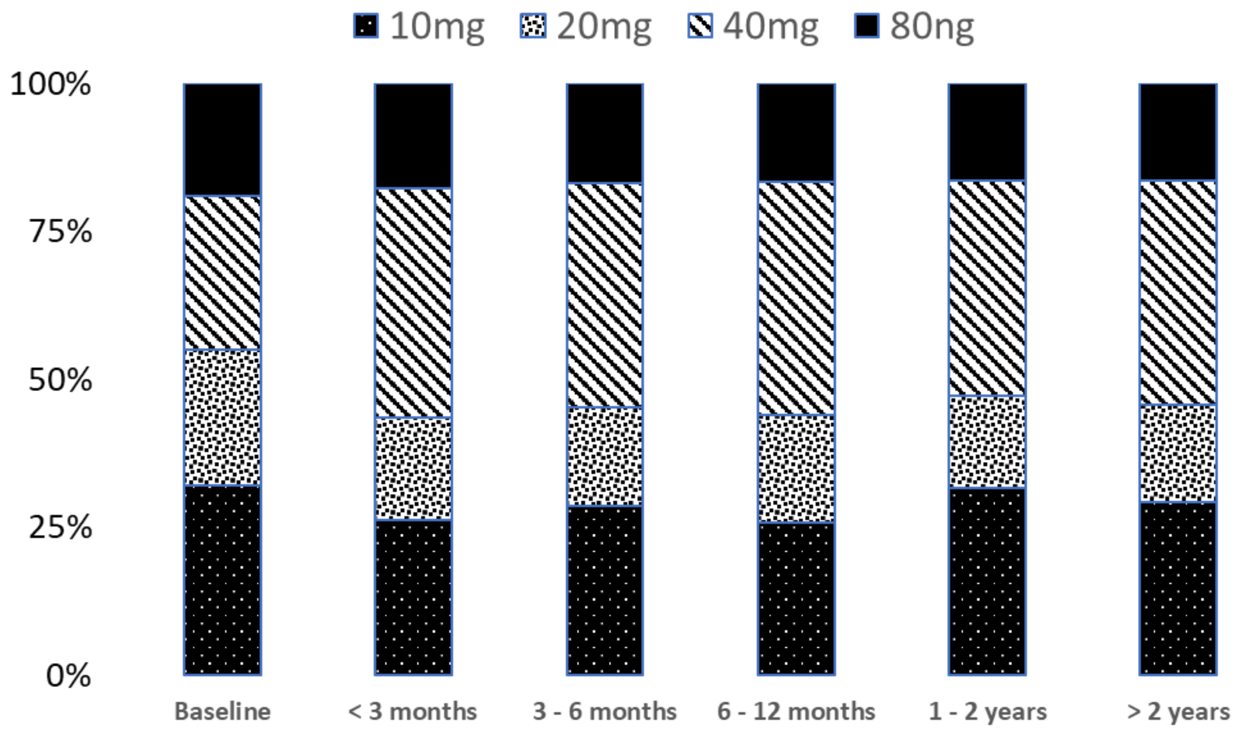

Supplement: Supplementary file 1 [file Data_Sheet_1.PDF]
